# Evaluation of a lateral-flow nanoparticle fluorescence assay for TB infection diagnosis

**DOI:** 10.5588/ijtld.21.0391

**Published:** 2021-11-01

**Authors:** F. Stieber, J. Howard, D. Manissero, J. Boyle, N. Ndunda, J. Love, M. Yang, A. Schumacher, R. Uchiyama, S. Parsons, C. Miller, H. Douwes, Z. Mielens, T. Laing, V. Nikolayevskyy

**Affiliations:** 1 Qiagen Inc, Germantown, MD, USA; 2 Qiagen Manchester Ltd, Manchester, UK; 3 Former Qiagen employee; 4 Ellume Limited, East Brisbane, QLD, Australia; 5 Imperial College, London, UK

**Keywords:** TB infection, QuantiFERON, QIAreach QFT, IGRA, validation

## Abstract

**BACKGROUND:**

Programmatic management of TB infection is a critical component of the WHO End TB Strategy. Interferon-gamma release assays (IGRAs) overcome some limitations of the tuberculin skin test, but implementation of IGRA testing in low-resource settings is challenging.

**METHODS:**

In this feasibility study, we evaluated performance of a novel digital lateral-flow assay, the QIAreach® QuantiFERON® TB (QIAreach-QFT) test, against the QuantiFERON®-TB Gold Plus (QFT-Plus) assay. A population with a mix of risk factors for TB infection (111 donors) were sampled over multiple days. A total of 207 individual blood samples were tested according to the manufacturer’s instructions.

**RESULTS:**

The overall percentage agreement was 95.6% (two-sided 95% CI 91.8–98), with a positive percentage agreement (i.e., sensitivity) of 100% (95% CI 94.7–100) and a negative percentage agreement (i.e., specificity) of 95.6% (95% CI 90.6–98.4). All QFT-Plus positive specimens with TB1-Nil and TB2-Nil values less than 1 IU/ml tested positive on QIAreach-QFT.

**CONCLUSIONS:**

QIAreach QFT is a deployable, accurate testing solution for decentralised testing. It has the potential to overcome key hurdles for TB infection screening in high-burden settings thus helping to achieve the WHO End TB programme goals.

## Abstract (FR)

**CONTEXTE:**

La prise en charge programmatique de l’infection tuberculeuse est une composante essentielle de la stratégie de l’OMS pour mettre fin à la TB. Les tests de détection de l’interféron gamma (IGRA) pallient certaines limites du test d’intradermoréaction à la tuberculine, mais l’utilisation des tests IGRA en cas de faibles ressources est difficile.

**MÉTHODES:**

Dans cette étude de faisabilité, nous avons évalué les performances d’une nouvelle test numérique de flux latéral (QIAreach® QuantiFERON® TB [QIAreach-QFT]) par rapport au test QuantiFERON®-TB Gold Plus (QFT-Plus). Des prélèvements ont été effectués sur plusieurs jours chez une population ayant divers facteurs de risque d’infection tuberculeuse (111 donneurs). Au total, 207 échantillons sanguins individuels ont été testés conformément aux instructions du fabricant.

**RÉSULTATS:**

Le pourcentage de concordance globale était de 95,6% (IC 95% bilatéral 91,8–98), avec un pourcentage de concordance positive (c.-à-d., sensibilité) de 100% (IC 95% 94,7–100) et un pourcentage de concordance négative (c.-à-d., spécificité) de 95,6% (IC 95% 90,6–98,4). Tous les échantillons positifs par QFT-Plus avec des valeurs TB1-Nil et TB2-Nil inférieures à 1 UI/ml ont été testés positifs par QIAreach-QFT.

**CONCLUSIONS:**

QIAreach QFT est un test fiable, à même d’être déployé afin de décentraliser le processus de dépistage. Il peut pallier les principaux inconvénients du dépistage de l’infection tuberculeuse dans les pays à forte incidence, et peut ainsi contribuer à atteindre les objectifs de la stratégie de l’OMS pour mettre fin à la TB.

Almost 25% of the world population is estimated to have TB infection, and about 5–10% of this population will progress to active TB disease during their lifetimes.^1^,^2^ Programmatic management of TB infection is thus a critical component of the global End TB Strategy, which focuses on TB elimination^3^–^6^ and aims to achieve a 90% reduction in TB incidence and a 95% reduction in TB mortality by 2035.^7^,^8^ For decades, TB control programmes in high-burden countries have focused exclusively on active TB case detection; however, we know from multiple modelling studies that TB elimination can only be achieved through comprehensive strategies directed toward integrated TB infection management with adequate active TB management.^2^

The tuberculin skin test (TST) and interferon-gamma release assays (IGRAs), such as QuantiFERON^®^-TB Gold Plus (QFT-Plus; Qiagen, Hilden, Germany) and T-SPOT.*TB* (Oxford Immunotec, Abingdon, UK), are the main diagnostic tests for TB infection.^3^,^4^,^9^–^12^ Both are indirect tests that measure the cell-mediated immune response to TB. The IGRA tests use specific peptide antigens that simulate mycobacterial proteins. These proteins, early secreted antigenic target 6 (ESAT-6) and culture filtrate protein 10 (CFP-10), are absent from all bacille Calmette-Guérin (BCG) strains and from most non-tuberculous mycobacteria, with the exception of *M. kansasii*, *M. szulgai* and *M. marinum*.^13^ Individuals infected with *M. tuberculosis* complex organisms usually have lymphocytes in their blood that recognise these and other mycobacterial antigens. This recognition process involves the generation and secretion of the cytokine, interferon-gamma (IFN-γ). The detection and subsequent quantification of IFN-γ forms the basis of the IGRA test.

The TST and IGRAs have several limitations and challenges. Although the TST is still widely utilised, particularly in developing countries, it requires two visits by the patient several days apart and the interpretation of the results is subjective, needing an induration reading in mm (which is prone to intraand inter-individual variation).^14^ IGRAs require highly skilled laboratory professionals and sophisticated laboratories to run the assays. There is therefore an urgent need for the development of point-of-care or near-patient diagnostic tests that can be deployed at the more decentralised levels of care, where patients have their initial contact with the health system.^15^

QIAreach^®^ QuantiFERON^®^-TB (QIAreach QFT; Qiagen) was developed to improve workflow efficiency and ease of operation in high TB burden, low-resource settings that have little to no access to sophisticated laboratories. QIAreach QFT is a novel digital lateral-flow test that uses patented nanoparticle fluorescence technology to qualitatively measure IFN-γ in plasma via its unique digital detection cartridge (eStick, Figure 1) and 8-port eHub design. The flexible design allows for low to higher volume testing of up to 24 tests per eHub per hour. While QFT-Plus utilises a set of four blood collection tubes (BCTs) (Nil, TB1, TB2 and mitogen), QIAreach QFT uses only one BCT, which is the equivalent of the TB2 tube. The single BCT contains antigens optimised to stimulate both CD4^+^ and CD8^+^ T-cells; this is the most sensitive tube in the QFT-Plus assay.^12^,^16^,^17^

**Figure 1:**
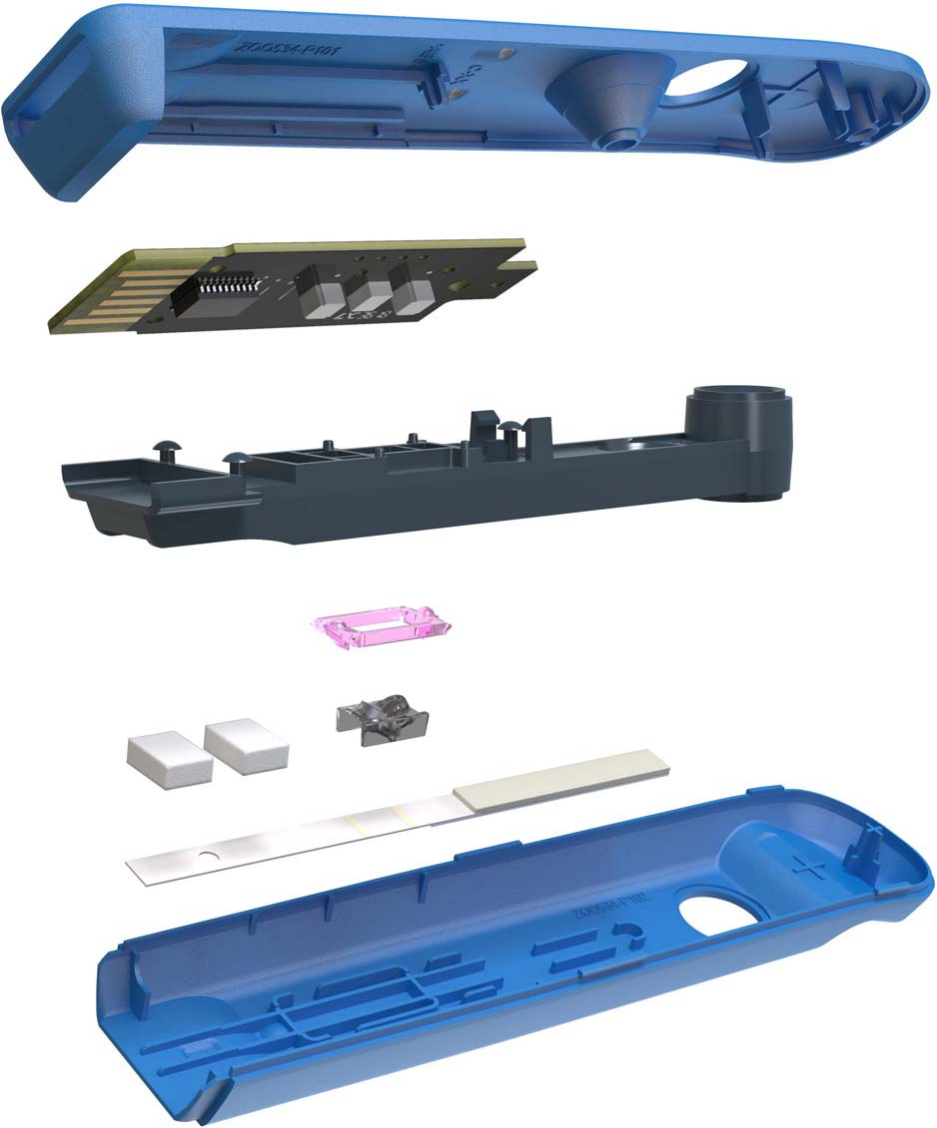
QIAreach QFT digital detection cartridge (eStick). Exploded QIAreach QFT eStick. Casing (blue) contains lateral flow assay strip with optoelectronic technology and a microprocessor that converts a fluorescent signal into a qualitative readout for the detection of interferon-gamma in the plasma sample that has been stimulated by peptide antigens that are associated with M. tuberculosis.

The aim of the present study was to assess the performance of QIAreach QFT against the QFT-Plus assay and ensure it meets clinical performance and user needs.

## METHODS

### Study samples

Whole-blood specimens were collected prospectively from 111 consenting subjects with and without varying risk factors for TB infection based on a self-assessment questionnaire. Baseline clinical, demographic and epidemiological data were collected (Table 1). All subjects were recruited locally within the Washington DC metropolitan region (USA), and all blood was processed and samples tested at QIAGEN Sciences Inc. in Germantown, MD, USA. Exclusion criteria included <18 years of age and not providing informed consent; otherwise subjects were considered eligible for this study. A subgroup of consenting subjects provided multiple specimens, with each specimen collected on a different day, for a total of 207 samples for use in the evaluation. Of all volunteers recruited for the study, 52 donors provided a single blood sample, 31 donors provided two samples, 19 donors provided three samples and 9 donors provided four samples. The time interval between multiple collections ranged from 1 week to 6 months

**Table 1:** Baseline clinical, epidemiologic and demographic characteristics of study participants (n = 111)

Characteristics	*n/N* (%)	Median [IQR]
Sex		
Male	61/102 (59.8)	
Female	41/102 (43.2)	
Age, years		50 [25–71]
US-born	72/102 (70.6)	
Foreign-born	30/102 (29.4)	
Medical history		
Active TB diagnosis at present/in the past	9/109 (8.3)	
On treatment for active or latent TB	9/104 (8.7)	
Positive tuberculin skin test result	9/97 (9.3)	
Positive IGRA test result	10/110 (9.1)	
Recent contact with a suspected/confirmed active TB case	9/104 (8.7)	
Recent travel/residence in a high TB incidence area	9/97 (9.3)	
BCG-vaccinated	24/100 (24.0)	
Tested positive for HIV	9/110 (8.2)	
Epidemiology		
History of incarceration	9/96 (9.4)	
Homeless	9/90 (10.0)	
Healthcare worker having face to face contact with patients with suspected or confirmed active TB	9/109 (8.3)	

IQR = interquartile range; IGRA = interferon-gamma release assay; BCG = bacilli Calmette-Gu érin.

### Sample collection and storage

Specimens were collected and processed based on standard procedures used by manufacturers and vendors; blood specimens for this study were collected using lithium heparin BCTs. The blood was then transferred aseptically into the four QFT-Plus tubes (Nil, TB1, TB2 and mitogen) and at least one additional TB2 tube, which is the functional equivalent of the QIAreach QFT BCT. BCTs were incubated at 37°C for 16–24 hours, after which tubes were centrifuged and plasma was harvested from the BCTs, or alternatively plasma was harvested directly from the BCTs without centrifugation. If more than one TB2 tube was collected per subject, then the plasma from individual TB2 tubes was transferred into a single tube, mixed by inversion, and subsequently aliquoted into separate tubes prior to testing.

### QFT-Plus enzyme-linked immunosorbent assay

Plasma specimens were tested according to the manufacturer’s guidelines, and the optical density (OD) values read. OD values from the QFT-Plus enzyme-linked immunosorbent assay (ELISA) assay were converted to IFN-γ IU/mL and the results were calculated using QFT-Plus Software v2.71.2. The subject result output of positive/negative/indeterminate was determined and used for analysis.

### QIAreach QFT test

QIAreach QFT is a qualitative in vitro diagnostic test using a peptide cocktail simulating ESAT-6 and CFP-10 proteins to stimulate cells in heparinised whole blood. Detection of IFN-γ by nanoparticle fluorescence is used to identify in vitro responses to these peptide antigens that are associated with *M. tuberculosis* infection. QIAreach QFT is a semi-automated, indirect test for *M. tuberculosis* infection (including disease) and is intended for use in conjunction with risk assessment, radiography, and other medical and diagnostic evaluations. The QIAreach test was performed according to the manufacturer’s instructions in a blinded manner, i.e., operators did not have access to QFT-plus results.

Briefly, the digital detection cartridge (eStick) was connected to a power source (eHub). Diluent buffer was first added to the processing tube, and reconstituted anti-IFN-γ nanoparticle conjugate. Patient plasma from the stimulated QIAreach QFT BCT was then harvested and added to the processing tube and mixed to resuspend the conjugate. The sample was then transferred to the eStick sample port, and the test started automatically. IFN-γ responses were measured via nanoparticle fluorescence. The eStick contains optoelectronic technology and a microprocessor that converts a fluorescent signal into a qualitative readout (Figure 1). Upon test completion, positive or negative test results from the eStick firmware are reported on the OLED (organic light-emitting diode) display from each connection port of the eHub. When using the optional manufacturer’s software to backup or transfer test results, test data are automatically transferred to an attached computer using the eHub connection port, thus providing an integration into a laboratory information system.

Standard test duration (time to result, TTR) for QIAreach QFT-negative samples is 20 minutes. For QIAreach QFT-positive samples, an internal firmware algorithm allows an early positive result to be determined if the IFN-γ response is above a predetermined threshold.

### Statistical analysis

The overall percentage agreement (OPA) was determined using QFT-Plus as the reference method. The positive percentage agreement (PPA; sensitivity) and negative percentage agreement (NPA; specificity) were calculated, along with the corresponding exact two-sided 95% confidence interval (CI) using QFT-Plus as a reference. Correlation analysis was performed and graphs constructed using GraphPad Prism v9.0.2 (GraphPad Inc, San Diego, CA, USA).

## RESULTS

### Samples

Of 207 samples tested in the study, 206 were used in the analysis for the performance agreement of QIAreach QFT against QFT-Plus. One subject was excluded after a QFT-Plus test handling error was detected.

**Figure 2:**
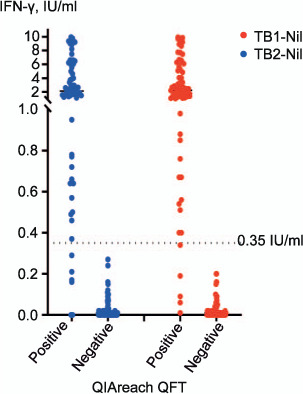
Performance of QFT-Plus antigen blood collection tubes vs. QIAreach QFT. IFN-γ = interferon-gamma.

Comparison of the QFT-Plus TB1 and TB2 antigen BCTs (Nil-subtracted) responses with the positive and negative QIAreach QFT test results showed a high agreement between qualitative test results and the range of IU/mL values within the study (Figure 2, Table 2). All 68 samples which tested positive using QFT-Plus also tested positive on QIAreach QFT, resulting in a PPA of 100% (95% CI 94.7–100), while 129/135 QFT-Plus-negative specimens also tested negative on QIAreach QFT (NPA 95.6%, 95% CI 90.6–98.4%). OPA between tests was high (95.6%, 95% CI 91.8–98.0).

**Table 2:** QIAreach QFT performance vs. QFT-Plus

	Frequency *n/N*	Agreement %	Upper 95% CI %	Lower 95% CI %
OPA	197/206	95.6	98.0	91.8
PPA	68/68	100.0	100.0	94.7
NPA	129/135	95.6	98.4	90.6

CI = confidence interval; OPA = overall percentage agreement; PPA = positive percentage agreement; NPA = negative percentage agreement.

**Figure 3:**
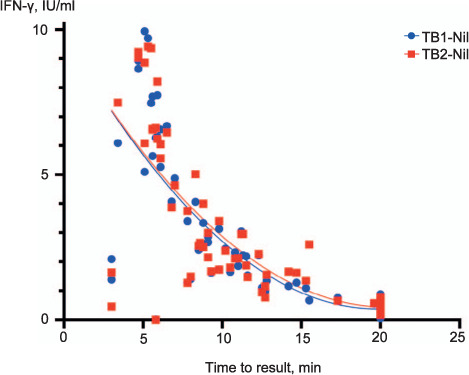
QIAreach QFT-positive time to result correlation with QFT-Plus (IU/mL). IFN-γ =interferon-gamma; QFT=QuantiFERON.

In 10 QFT-Plus-positive specimens (4.9%), both the TB1-Nil and TB2-Nil values were <1 IU/mL and all returned a positive QIAreach QFT result (Figure 2). The QIAreach QFT TTR values for positive samples were compared to the corresponding IFN-γ values of the QFT-Plus TB1 and TB2 antigen tubes, demonstrating good correlation between TTR and IFN-γ concentrations detected using QFT-Plus (Figure 3). Shorter TTRs were associated with higher IFN-γ values in both TB1 and TB2 tubes.

## DISCUSSION

This is the first study assessing the diagnostic performance of QIAreach QFT against QFT-Plus. Of the 206 QFT-Plus samples tested, we found a 100% sensitivity (PPA) and 95.6% specificity (NPA). All 10 QFT-Plus-positive specimens with TB1-Nil and TB2-Nil values under 1 IU/ml tested positive on QIAreach-QFT. Of the 9 non-concordant results, 3 were QFT-Plus-indeterminate results with Nil tube values >10 IU/mL that were called QIAreach QFT-positive and 6 were QIAreach QFT-positive with QFT-Plus negative result values below the 0.35 IU/mL cut-off; all non-concordant results were investigated. There was no evidence of endogenous analytical interference at the concentrations listed above.

The QIAreach QFT assay leverages the high sensitivity of the QFT-Plus TB2 tube. A study published by Barcellini et al. evaluated the performance of each of the QFT-Plus tubes (TB1 and TB2). The authors observed a significant difference in IFN-γ response between the two tubes, showing that the TB2 tube has the highest sensitivity of the two.^18^ A meta-analysis of 15 studies published by Sotgiu et al. showed that the QFT-Plus assay had a pooled sensitivity of 94% for active TB patients and a pooled specificity of 96% in healthy individuals.^17^ It was also noted that significant correlation was observed between the QIAreach QFT TTR and IU/mL responses of QFT-Plus-positive samples.

**Figure 4:**
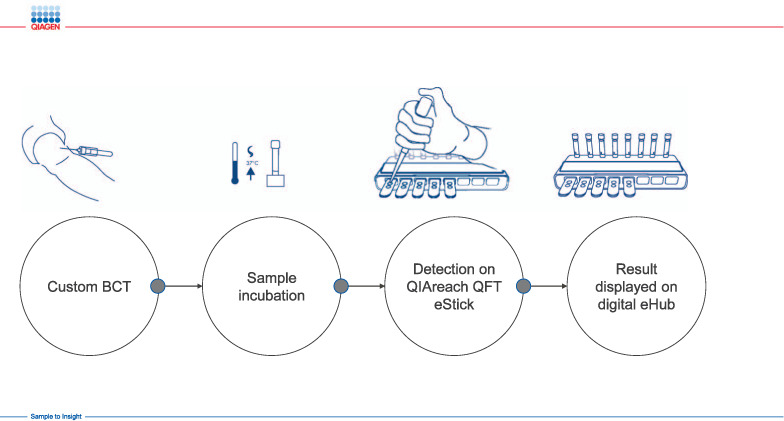
QIAreach assay workflow. The QIAreach QFT workflow is fast, simple and easy and has four steps: 1) collect blood into a single QIAreach QFT BCT; 2) incubate BCT to stimulate an in vitro TB immune response; 3) perform the QIAreach QFT digital detection assay using a disposable cartridge (eStick) powered by a multi-port reader (eHub); 4) read result on eHub display in 3–20 minutes. BCT = blood collection tube.

QIAreach QFT is simple to perform, providing rapid, precise results in four steps (Figure 4) and demonstrating high sensitivity (100% in our study) compared to established QFT-plus methodology.^17^,^18^ To note, this novel digital lateral flow technology provides sensitivity equivalent to ELISA, as demonstrated by the 100% positivity in specimens with TB1-Nil and TB2-Nil values <1 IU/ml, which is superior to that reported for another lateral flow-based IGRA assay.^19^,^20^ By leveraging this novel digital lateral flow technology, QIAreach QFT eliminates the need for more complex ELISA-based technology currently affecting roll-out of IGRA tests in many settings.^1^,^6^,^7^.The simple design using a battery-operated rechargeable eHub allows for expanded near-patient testing in high-burden, low-resource environments, as minimal laboratory skills and infrastructure are required. This could support the expansion of IGRA testing and TB prevention as a programmatic component in higher-incidence settings thus helping to achieve the WHO End TB programme’s ambitious goals.^8^,^20^

Lack of direct head-to-head comparison between QIAreach and sputum culture, which made it impossible to determine the sensitivity of the assay for active TB detection, constitute one of the principal limitations of our study; however, high sensitivity vs. QFT-Plus demonstrated in our study could be indicative of a similarly high sensitivity for active TB. Other limitations include the relatively small sample size, single-centre study design and the use of low-incidence settings only.

The positive predictive value of IGRAs remains low, resulting in relatively high numbers needed to treat to prevent TB.^20^,^21^ The specificity of QIAreach QFT was only marginally lower than that of QFT-Plus in our study (95.6%), which suggests that the potential roll-out of the assay in decentralised settings is very unlikely to affect adversely number needed to treat. However, further evaluations that will analyse the performance of QIAreach in culture-confirmed patients and other populations in various settings, including high TB burden and low-resource environments, will be needed to identify its role and place for routine TB infection screening.

In conclusion, QIAreach QFT is a novel innovative technology that shows a high level of agreement with QFT-Plus but requires significantly less laboratory infrastructure and resources due to elimination of ELISA. It has the potential to overcome key hurdles for TB infection screening in high-burden, low-resource settings and could be considered a promising scalable solution for decentralised TB infection screening within local and national TB programmes.
